# Improved medication adherence and health literacy in parents of children with ADHD: Effects of a targeted educational program

**DOI:** 10.1016/j.rcsop.2025.100634

**Published:** 2025-07-09

**Authors:** Nafiseh Valaei Sharif, Peivand Ghasemzadeh, Niayesh Mohebbi, Sogand Ghasemzadeh

**Affiliations:** a Department of Pharmacoeconomics and Pharmaceutical Management, TeMS.C., Islamic Azad Univerity, Tehran, Iran; b Department of Clinical Pharmacy, Tehran University of Medical Sciences, Tehran, Iran; c Department of Psychology and Education of Exceptional Children, Faculty of Psychology and Education, University of Tehran, Tehran, Iran

**Keywords:** Attention deficit hyperactivity disorder, Health literacy, Medication adherence, Child

## Abstract

**Background:**

Although medication is important for children with Attention Deficit Hyperactivity Disorder (ADHD), medication adherence is low among parents. One of the key factors affecting medication adherence and disorder management is health literacy.

**Objective:**

This study aimed to assess the effectiveness of an educational program for parents of children with ADHD in improving medication adherence and health literacy.

**Methods:**

An educational program was developed that covered four main topics: general disorder information, treatment, parenting based on emotional needs, and basic health information. A total of 191 parents were screened and assigned 108 eligible participants to either the intervention group (*n* = 74) or the control group (*n* = 35). The intervention group received a two-month educational program delivered via messenger application, while the control group received general counseling. Medication adherence and health literacy were measured before and after the intervention. *t*-test, ANOVA, ANCOVA, and chi-square test were used to compare group differences.

**Results:**

Post-intervention, the intervention group showed significantly higher medication adherence (6.87 ± 1.36) than the control group (5.46 ± 1.45). The intervention group also demonstrated higher health literacy scores (82.34 ± 6.96) than the control group (72.15 ± 6.52). Covariance analysis revealed significant improvements in health literacy (F = 162.73, *p* < 0.001, eta squared = 0.657) and medication adherence (F = 40.41, p < 0.001, eta squared = 0.322) scores. A significant difference was found in medication adherence improvement based on economic status (*p* = 0.037) in the intervention group. However, no significant differences in medication adherence and health literacy were observed based on parental gender, education level, or insurance status.

**Conclusion:**

The educational program showed improvement in medication adherence and health literacy among parents of children with ADHD.

## Highlights

• The educational program demonstrated improvements in parents' health literacy and medication adherence for managing ADHD.
• Delivering the intervention through an educational program could be an effective and engaging method for parent education.
• Parents with medium income showed the most improvement in adherence, suggesting economic status may influence outcomes.
• Higher health literacy was associated with better medication adherence among parents.

## 1 Introduction

Attention deficit hyperactivity disorder (ADHD) is a common neurodevelopmental disorder characterized by inattention, hyperactivity, and impulsivity.^1^ While symptoms may change over time, research indicates that a significant proportion of individuals continue to experience core symptoms into adulthood, affecting approximately 2.58 % of adults globally^2^, ^3^, ^4^ and approximately 3.8 % in Iran.^5^ This persistence highlights the chronic nature of ADHD and the need for ongoing management from childhood through adulthood. The disorder impacts various aspects of life, including academic and occupational performance, social relationships, self-esteem, and risk-taking behaviors.^6^

The effects of ADHD extend to families, straining relationships, increasing parenting stress, and causing financial hardship.^7^^,^^8^ Costs associated with raising a child with ADHD are primarily driven by education, healthcare, and reduced parental income.^9^

Given ADHD's chronic nature and associated costs, pharmacological therapy is strongly recommended alongside non-pharmacological interventions to manage symptoms and improve outcomes.^10^ However, medication adherence rates among children and adolescents with ADHD are low due to multiple daily dosing, stigma, side effects and ineffectiveness.^11^^,^^12^ Studies show that within twelve months, stimulant medication adherence in children ranges from 50 % to 75 %, dropping below 50 % after three years. Many families stop medication after the first year.^13^, ^14^, ^15^ A survey in Iran found that nearly 78.8 % of caregivers did not strongly adhere to treatment.^16^

The World Health Organization (WHO) defines medication adherence as “the degree to which a person's behavior is consistent with the recommendations agreed with the health care provider”.^7^ Non-adherence leads to increased medical resource use, higher hospital admission rates, and more frequent emergency department visits, and can negatively impact patients' quality of life and functional abilities.^17^^,^^18^ By obtaining a deep understanding of the disorder, parents can overcome misconceptions and reduce barriers to adherence.^19^

The WHO's "Global Strategy on Digital Health 2020-2025"emphasizes the importance of digital technologies in promoting sustainable health systems and achieving universal health coverage. Digital health, or eHealth, uses information and communication technologies to manage, deliver, and optimize patient care and health services while empowering patients.^20^^,^^21^ A recent systematic review found that digital health interventions (DHIs) improve medication adherence among children and adolescents with acute or chronic ADHD and leads to positive outcomes for patients and their families (e.g., web-based interventions, m-Health, Telemedicine).^21^^,^^22^ DHIs help bridge gaps between patients and healthcare professionals, allowing for more frequent monitoring, communication, patient education, and assessments.^21^ As technology advances, these tools can be innovative approaches to improve health literacy and medication adherence, especially for people with chronic diseases. By supporting patient education, self-management, treatment options, clinical decision-making, and provider communication, digital health technologies can significantly improve healthcare delivery.^23^

Health Literacy is crucial in improving medication adherence, reflecting a patient's ability to receive, process, and understand basic health information and services needed for appropriate health decisions.^24^, ^25^, ^26^ People with lower health literacy, often lack knowledge about their condition, adhere poorly to medication regimens, undergo fewer examinations, and face higher hospitalization rates.^27^

As a primary goal of public health education and communication strategies,^25^ health literacy is essential for adequate disease control and prevention of adverse outcomes.^28^ According to WHO reporting and Iranian studies, health literacy levels in the general population are insufficient, as in other developed and developing countries.^29^^,^^30^ Therefore, developing accessible and effective strategies to improve medication adherence and health literacy could significantly reduce the clinical and financial burden of ADHD on children and their families.^31^

## 2 Aim

This study examined the effectiveness of a psychoeducational program in enhancing medication adherence and health literacy among parents of children and adolescents (6 to 12 years old) diagnosed with ADHD.

## 3 Methods

In November 2023, this study was conducted comprising three phases: participant screening and enrollment, parent training, and assessment of the psychoeducational program's impact.

### 3.1 Participants

A total of 191 parents were screened for eligibility. After applying inclusion and exclusion criteria and obtaining informed consent, 109 participants were enrolled in the study and allocated to the intervention group (*n* = 74) or control group (*n* = 35) through referrals from collaborating child and adolescent psychiatrists in Tehran from November 2023 to January 2024.

Participants were recruited through collaboration with child and adolescent psychiatrists. Healthcare professionals were contacted and provided with detailed information about the study. Upon review, those who agreed to participate assisted in the recruitment process. A thorough screening process was implemented to ensure participants met the study's inclusion and exclusion criteria. Eligible children were between 6 and 12 years old, diagnosed with ADHD and prescribed medication by a psychiatrist. This age group is primarily influenced by their parents, and the parents play an important role in administering medication. While this recruitment strategy ensured access to parents actively seeking resources, it may have introduced selection bias by favoring parents with higher engagement in their child's care. Efforts were made to screen participants thoroughly to ensure their suitability for the intervention while minimizing bias in recruitment.

Children who were unsuitable for medication treatment, had a primary diagnosis of pervasive developmental disorders, autism, or other psychoses, were illiterate, or failed to complete the questionnaire at the end of the intervention were excluded from the study.

**Fig. 1:**
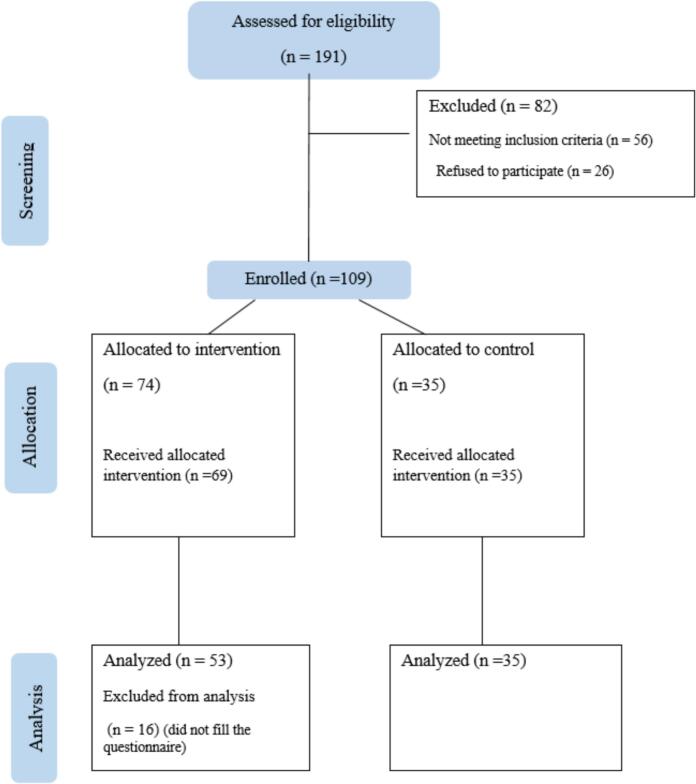
Participant flow diagram.

Participants were required to be the primary caregivers and legal guardians of children. All parents provided informed consent and were fully informed of the study conditions. Both intervention and control groups continued their regular psychiatrist visits and prescribed medication regimens. Fig. 1 illustrates the study's recruitment, allocation, and analysis processes.

In this study, participants were allocated into intervention and control groups based on availability and willingness to participate. While random assignment was not feasible due to practical constraints, groups were matched based on key characteristics such as child age, gender, and ADHD severity to minimize baseline differences. While the intervention group was larger (*n* = 74) compared with the control group (*n* = 35), this disparity occurred as a result of practical issues in participant recruitment, specifically meeting the inclusion criteria within the study timeline. Despite these differences, steps were taken to minimize potential biases. Participants were matched on key demographic and clinical characteristics, such as child age, gender, and ADHD severity, to ensure comparability at baseline. Moreover, statistical techniques such as ANCOVA were employed to adjust for potential confounding effects, thus ensuring the validity of the findings. Similar approaches in prior research have shown that results from studies with unequal group sizes remain robust when appropriate statistical adjustments are applied.^32^^,^^33^ Therefore, it is considered that, despite these limitations, the findings contribute meaningful insights into the potential efficacy of the educational intervention.

### 3.2 Procedure

Following participant screening and consent, participants were allocated to either the intervention or control group while matching on key demographic and clinical characteristics. Randomization was not performed due to practical constraints in recruitment. Both groups provided online written informed consent while remaining unaware of the educational content received by the other group. The sample size was calculated by using GPower's a priori sample size calculator, aiming for a one-way analysis of variance with a significance level (alpha) of 0.05, study power of 80 %, and anticipated effect size (Cohen's d) of 0.7. additional 10 % included to account for potential dropouts.

### 3.3 Intervention group

To improve medication adherence and ADHD-related health literacy among parents, a two-month comprehensive educational program was implemented. This educational program was conducted based on similar literature and particularly for this research. A dedicated channel was created on a popular messaging app to distribute all educational materials and content to the intervention group.

The intervention group received daily expert-guided educational content in various formats, including animated motion graphics, designed posts, and brief textual resources. These materials provided in-depth information about hyperactivity disorder and strategies to improve medication adherence. To encourage engagement and ensure attention to the educational content, daily short questions, polls, and quizzes with brief answers were incorporated into the channel. This interactive approach aimed to promote active participation, deepen understanding, and reinforce learning among parents in the intervention group.

### 3.4 Expert-guided content

Educational content incorporating key psychoeducation elements, as outlined by Sarkhel, Singh, and Arora,^34^ was designed to include:

• ADHD causes
• Common signs and symptoms
• Early relapse or recurrence identification
• Effective coping strategies
• Treatment options
• When and how to seek professional help
• Importance of treatment adherence
• Long-term course and outcomes of ADHD
• Addressing misconceptions and stigma

Materials were developed in consultation with clinical pharmacy and psychology experts to ensure accuracy. Additional input to refine the content was provided by clinical pharmacists.

The training program covered four main sections in Persian:

### 3.5 Part I: ADHD introduction

This section provided general information on ADHD prevalence, causes, signs, and symptoms. It explained the neurodevelopmental nature of ADHD and brain function differences between children with and without ADHD. The potential consequences of untreated ADHD on quality of life were also discussed.^6^ ADHD was approached in this study as a neurodevelopmental condition characterized by variability in symptom severity and presentation over time. While efforts were made to avoid framing ADHD as a debilitating mental disorder to reduce potential stigma, it is acknowledged that, for many children and their families, the condition can be significantly challenging, affecting daily functioning, academic performance, and overall well-being. Therefore, the educational program was designed to provide balanced information, addressing both the difficulties associated with ADHD and strategies for effective management and support.

### 3.6 Part II: Pharmacotherapy

This section aimed to inform parents about ADHD treatment options, including different medication types and their mechanisms of action. Common side effects that can lead to non-adherence were addressed, and practical management solutions were provided. The program also tackled parental misconceptions about medication such as fears of brain degeneration or ineffectiveness and common barriers to medication adherence. Information on proper medication administration was implemented for both stimulant and non-stimulant medications, including optimal timing to minimize side effects.^10^^,^^35^, ^36^, ^37^, ^38^

### 3.7 Part III: parenting and emotional needs

Materials and tips based on the emotional needs of parents with children with ADHD were presented to improve parenting and coping skills.

### 3.8 Part IV: general health information

This section covered various health topics, including healthy diet for parents and children, dietary recommendations for children with ADHD, when to consult a doctor or psychiatrist, the pharmacist's role in counseling, understanding medication brochures, mental health tips, common medical orders (e.g., fasting for lab tests, medication administration intervals) Throughout the program, parents could ask questions directly from program administrators, receiving answers and personalized counseling from specialists.

### 3.9 Interactive engagement (Polls and Feedback)

To encourage engagement and better understand participants' beliefs and challenges, brief daily polls and quizzes were incorporated into the messaging app. These anonymous polls covered topics such as concerns about medication side effects, beliefs about stimulant addictiveness, reasons for non-adherence, and perceived barriers to following treatment plans. Poll results were used to tailor follow-up educational content and reinforce key messages.

### 3.10 Control Group

Parents in the control group maintained their usual care routines and psychiatrist appointments without participating in the educational program. After the intervention phase, the same educational content was offered to them through a dedicated channel.

### 3.11 Measures

Data were collected using online questionnaires at two time points: baseline (T1), conducted during the week prior to the start of the educational program in November 2023, and follow-up (T2), completed within two weeks (10–14 days) after the end of the two-month intervention period in January 2024.

#### 3.11.1 Health literacy

The Health Literacy for Iranian Adults (HELIA) tool, which was validated for the Iranian population by Montazeri et al. was used to assess participants' health literacy.^39^ This 33-item questionnaire assessed health literacy across five dimensions: access, reading, understanding, appraisal, and decision. The total health literacy score ranged from 0 to 100. Health literacy was categorized as high (84.1–100), adequate (50.1–84), or insufficient (<50).^40^

Medication Adherence.^1^

The Persian version of the Morisky Medication Adherence Scale (MMAS-8), previously validated in Iran for chronic diseases, was employed. The MMAS-8 includes seven yes/no questions and one five-point Likert scale item, with each item assessing specific medication-taking behaviors. Total MMAS-8 scores ranged from 0 to 8, with scores of 8 indicating high adherence, 6 to 8 indicating medium adherence, and below 6 indicating low adherence.^41^^,^^42^

### 3.12 Statistical analysis

Statistical analysis was conducted using SPSS™ version 26. Descriptive statistics, means, and standard deviations were calculated. *t*-test was used for continuous variables and chi-square tests for categorical variables between the intervention group and the control group. Inferential statistical methods, ANOVA and ANCOVA were then applied. The significance level was 0.05.

## 4 Results

### 4.1 Baseline demographic characteristics

Of the 191 parents who completed the screening questionnaire, 56.5 % met the screening criteria. Fifty-six parents were excluded based on inclusion criteria, and 27 were excluded for declining to participate in the program.

Among the 191 parents initially enrolled in the study, post-intervention assessments were not completed by 28 % of participants in the intervention group. The main reasons for drop-out were scheduling conflicts (and difficulty attending to the program's channel) (21 %). A comparison of demographic and clinical characteristics between completers and non-completers indicated no significant differences, suggesting that the drop-out group was not systematically different from the completers. To address attrition bias, an intention-to-treat analysis was conducted in which all participants were included, and multiple imputations were applied to handle missing data.

Table 1 presents the demographics of children and their parents. Mothers made up over 94 % of participants in each group. In both groups, more than 70 % of parents had an academic education (bachelor's degree or higher), and over 90 % were employed. Boys constituted the majority of children with ADHD, comprising more than 85 % in the control group and 70 % in the intervention group. The mean age was approximately 9 years for children and 38 years for parents.

**Table 1:** Patient and parent demographic characteristics.

Variable	Category	Control group		Intervention group		P -Value
		n	N%	N	N%	
Participants	Mothers	33	94.3	51	96.2	0.66
	Fathers	2	5.7	2	3.8	
Parents' educational status	Non-academic	11	31.4	15	28.3	0.82
	Bachelor's degree	15	42.9	27	50.9	
	Master's degree	6	17.1	6	11.3	
	PhD or Higher	3	8.6	5	9.4	
Parental work status	Homemaker	21	60	33	62.3	0.62
	Employed	12	34.3	19	35.8	
	Unemployed	2	5.7	1	1.9	
Previous marriage history	Yes	33	94.3	51	96.2	0.669
	No	2	5.7	2	3.8	
Patient's gender	Female	5	14.7	15	28.3	0.14
	Male	30	85.7	38	71.7	
Medication	Stimulants	17	48.57	28	54.90	0.851
	Non-stimulants	3	8.57	6	11.76	
	Risperidone	13	37.14	17	33.33	
	Other	2	5.71	2	3.92	
Insurance	Yes	32	91.4	48	90.6	0.89
	No	3	8.6	5	9.4	
Economic status	Low	6	17.1	10	18.9	0.69
	Medium	15	42.9	18	34	
	high	14	40	25	47.2	
Patient's age (years)	Mean ± SD	9.88 ± 2.54		9.94 ± 2.67		0.920
Parent's age (years)	Mean ± SD	38.22 ± 5.46		38.13 ± 5.43		0.93
Parents' satisfaction	Mean ± SD	____		8.09 ± 1.60		___

*Satisfaction was only measured in the intervention group after the program.

### 4.2 Scores of health literacy and medication adherence

**Fig. 2:**
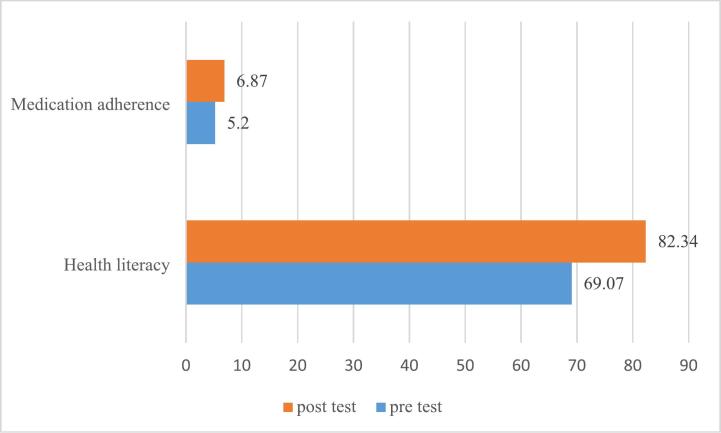
Health literacy and medication adherence score of the intervention group at baseline and end of the education program.

Table 2 summarizes the mean scores for health literacy and medication adherence of parents in both groups at T1 and T2. Initially, medication adherence scores were low for parents in both groups. However, in the post-test stage, the intervention group's score increased to a medium level of medication adherence (6.87 ± 1.36, *p* < 0.05). Health literacy scores were similar for both groups in the pre-test. By the end of the program, the intervention group showed an increase in health literacy levels comparing the control group (82.34 vs 72.15). Fig. 2 shows the health literacy and medication adherence scores of the intervention group before and after the educational program.

**Table 2:** Mean health literacy and medication adherence scores for parents in both groups.

Variable	Group	Pre-test	Post-test	*P*-Value
Health literacy	Control	8.16 ± 72.09	6.52 ± 72.15	0.923
	Intervention	9.08 ± 69.07	6.96 ± 82.34	0.001
Medication adherence	Control	1.28 ± 5.49	1.45 ± 5.46	0.849
	Intervention	1.45 ± 5.20	1.36 ± 6.87	0.001

### 4.3 Impact of educational program and type of medication on medication adherence

The results indicated that the educational program significantly improved medication adherence among parents of children with ADHD, with a more pronounced effect observed in those prescribed stimulants. In the intervention group, adherence scores increased significantly from pre- to post-test for both stimulants (from 4.60 to 6.95) and the other type of medications (from 5.87 to 6.79), with both comparisons reaching statistical significance (*p* < 0.001). In contrast, no significant changes were observed in the control group for either medication type. ANCOVA results further confirmed significant main effects of group and medication type, as well as a significant interaction between the two, suggesting that the intervention's effectiveness was moderated by the type of medication prescribed. These findings are presented in supplementary tables S1 and S2.

### 4.4 Impact of educational programs on health literacy and medication adherence

ANCOVA was conducted to evaluate the effect of the educational intervention on health literacy and medication adherence. The results revealed a significant difference in health literacy between the intervention and control groups. The main effect on health literacy (F = 162.73, *p* < 0.05, η^2^ = 0.657) and medication adherence (F = 45.10, p < 0.05, η^2^ = 0.322) for the intervention group was statistically significant. An Eta squared value (η^2^) of 0.657 indicated that 65.7 % of the variance in health literacy and 32.2 % in medication adherence was explained by the educational program.

### 4.5 Comparison of medication adherence and health literacy improvements: parental role, education, income, and insurance status

The results of Levene's test (*p* > 0.05) confirm the homogeneity of variances in both groups. Analyses showed no statistically significant differences in the change between post-test and pre-test scores for medication adherence or health literacy based on parental gender, education levels or insurance status in either group. These findings are presented in supplementary tables S3, S4 and S5.

However, a significant difference was observed in the change in medication adherence scores intervention group participants based on income levels (*p* = 0.037). Specifically, participants in the medium-income group demonstrated the greatest improvement (mean increase = 2.25), compared to those in the low-income (0.08) and high-income (1.61) groups. These findings are detailed in Table 3.

**Table 3:** Medication adherence and health literacy by income.

Variable	Group	Economic Status	Mean	SD	F	P-value
Medication adherence	Control	Low	0.12	0.34	0.322	0.727
		Medium	−0.16	1.25		
		High	0.05	0.51		
	Intervention	Low	0.08	1.29	3.51	0.037
		Medium	2.25	1.56		
		High	1.61	1.30		
Health literacy	Control	Low	2.41	3.81	1.72	0.194
		Medium	0.26	4.17		
		High	−1.15	3.79		
	Intervention	Low	12.75	5.04	1.94	0.153
		Medium	15.52	6.72		
		High	11.85	5.95		

### 4.6 Correlation between health literacy and medication adherence

Table 4 shows a significant positive correlation between health literacy and medication adherence in the post-test, both overall and within the intervention group. This indicates that higher medication adherence is associated with increased health literacy, and conversely, improved health literacy correlates with better medication adherence.

**Table 4:** Correlation between health literacy and medication adherence.

Variables	Group	Test	Correlation Coefficient	P-Value
Correlation of medication adherence with health literacy	Control	Pre-test	0.172	0.323
		Post-test	0.071	0.687
	Intervention	Pre-test	0.096	0.494
		Post-test	0.319	0.020

## 5 Discussion

The educational program for parents had four sections: ADHD knowledge, treatment, parenting based on basic emotional needs, and general health tips. This approach aligns with previous studies demonstrating that psychoeducation improves ADHD awareness, treatment knowledge, and medication adherence.^19^^,^^43^, ^44^, ^45^, ^46^ With the difference is that all of the educational contents were presented in a messenger application.

The findings align with previous research indicating low medication adherence among children with ADHD, which is a significant problem.^47^^,^^48^ Initially, the mean medication adherence score was low for parents in both groups. This could be due to parent's beliefs, adverse effects, multiple dosing and, addictiveness of stimulants according to polls conducted during the intervention.

After the psychoeducation program, the intervention group showed significant increases in average health literacy and medication adherence scores compared to the control group. This outcome is consistent with earlier randomized clinical trials. Bai et al.'s (2015) RCT found that psychoeducation positively affected medication adherence and parents' ADHD knowledge.^19^ Zheng et al.(2020) demonstrated that comprehensive training programs for parents and teachers improved ADHD understanding and medication adherence.^44^ Monastra ‘s neuro-educational program for ADHD children's parents (2005) addressed treatment barriers and concerns, resulting in 95 % of patients following medical recommendations, starting pharmacological treatment, and continuing medication for a two-year follow-up period.^45^

This study demonstrated a significant positive relationship between increased health literacy and medication adherence, with higher health literacy corresponding to better medication adherence in parents. Health literacy refers to people's ability to find, understand, and use health information and services to make informed decisions. Improving an individual's knowledge about a disease leads to a better understanding and application of information in managing their health condition.^49^ Limited health literacy is associated with poor patient-physician communication, inadequate disease management skills, suboptimal treatment outcomes, and non-compliance with drug regimens.^39^^,^^50^ Consequently, improving health literacy can be an effective approach to improving self-management skills, medication adherence, and overall quality of life for families with children with ADHD.^27^

However, inadequate health literacy is associated with low socioeconomic status and poor health,^51^ even though highly educated people may also have poor health literacy skills.^52^ However, this study showed no significant differences between parents' health literacy and their education. This contrasts with Chris van der Heide, Wang, Droomers et al.'s (2013) findings, which suggested that health literacy partially mediates the relationship between low education and poor health status. They proposed that improving health literacy might help reduce health inequalities by explaining the mechanism linking low education to poor health.^53^

Social and economic factors, including income, typically affect medication adherence,^54^ this study found no significant relationship between medication adherence and parents' income. This result diverges from Safavi and Saberzadeh (2019)^16^ and Zheng et al. (2020)^44^ but aligns with Rieppi et al. (2002).^55^ Safavi and Saberzadeh ‘s study (2019) on factors related to medication adherence in children with ADHD revealed negative correlations between adherence and inattention scores in teacher reports and poor economic status. They also found positive correlations between adherence and a family history of psychiatric treatment, as well as the father's education level. In contrast with the present study, a significant relationship between medication adherence and a family history of ADHD was identified.

Zheng, Shen, Jiang et al. (2020) provided a training program for parents and teachers in their intervention group, covering ADHD behavior disorder, intervention strategies, and classroom management techniques. Their results showed a significant correlation between socioeconomic factors and medication adherence among parents of children with ADHD, with higher-income families demonstrating better adherence.^44^ In contrast, Rieppi, et al. (2002) found no significant difference in medication compliance between families with lower and higher income levels.^55^

Medication costs and insurance coverage are generally considered important factors in medication adherence for chronic diseases, particularly ADHD.^15^ For low-income individuals, medication cost is a well-known barrier to adherence.^56^ However, no significant relationship was found between insurance coverage and medication adherence in the present study. This outcome may be attributed to the relatively low cost of medication in Iran compared to other countries.^57^ Regarding ADHD medications, the cost of one mg of methylphenidate and lisdexamphetamine is 0.013$ and 0.004$ accordingly.^58^

### 5.1 Limitations

The use of self-reported data for medication adherence and health literacy may have introduced potential biases, including social desirability. The intervention, delivered through a single digital platform, might have varied in effectiveness across different platforms and user interfaces. The short duration of the intervention limited the ability to assess long-term adherence and sustained improvements in health literacy. In future interventions, longer follow-up periods may be incorporated to evaluate the durability of their effects. Additionally, the exploration of multimodal delivery platforms may enhance the intervention's reach and effectiveness.

The potential introduction of reminder bias must also be considered. The frequency and interactivity of the educational content delivery, including daily messages, polls, and quizzes, may have served as indirect reminders for adhering to medication regimens. Therefore, some of the observed improvements in adherence and health literacy might have been partially attributable to repeated engagement rather than the educational content alone. Future research should aim to isolate the effect of message frequency from content quality to better understand the specific mechanisms of improvement.

Changes in participants' medication regimens, such as dose adjustments, medication switching, or discontinuations, were not systematically tracked. These clinical changes could have potentially influenced adherence outcomes. Future studies should include medication tracking in order to better isolate the effects of educational interventions.

## 6 Conclusion

Study findings showed that the two-month psychoeducational program, delivered through online channels via a messaging app, demonstrated improvements in health literacy and medication adherence among parents of children with ADHD. These findings highlight the potential of online psychoeducational programs to equip parents with knowledge and skills for managing their child's ADHD effectively.

## CRediT authorship contribution statement

**Nafiseh Valaei Sharif:** Writing – review & editing, Writing – original draft, Project administration, Methodology, Data curation. **Peivand Ghasemzadeh:** Writing – original draft, Supervision, Conceptualization. **Niayesh Mohebbi:** Writing – review & editing, Supervision, Methodology. **Sogand Ghasemzadeh:** Supervision, Conceptualization.

## Declaration of competing interest

The authors declare that they have no known competing financial interests or personal relationships that could have appeared to influence the work reported in this paper.

## Appendix A Supplementary data

Supplementary material Supplementary tables and analyses Supplementary material

## Footnotes

1 The MMAS-8 Scale (U.S. Copyright Registration No. TX0008632533), content, name, and trademarks are protected by US copyright and trademark laws. Permission for use of the scale and its coding is required. A license agreement is available from MMAR, LLC, www.moriskyscale.com.
