# Supplementary material for: Improved medication adherence and health literacy in parents of children with ADHD: Effects of a targeted educational program
Source: Explor Res Clin Soc Pharm. 2025 Jul 9;19:100634. doi: 10.1016/j.rcsop.2025.100634 (PMC12284690; doi:10.1016/j.rcsop.2025.100634)
Supplement: Supplementary material — Supplementary tables and analyses [file mmc1.docx]

**Supplementary material**

**Table S1** Descriptive of medication adherence score based on group and medication type

| Variable | Group | Medication Type | N | Pre-test | | Post-test | | Paired t-test | |
| --- | --- | --- | --- | --- | --- | --- | --- | --- | --- |
|  |  |  |  | Mean | SD | Mean | SD | t | P-value |
| Medication Adherence | Control | Stimulants | 17 | 5.67 | 1.45 | 5.61 | 1.75 | 0.213 | 0.834 |
|  |  | Other types of medication | 18 | 5.31 | 1.12 | 5.32 | 1.10 | 0.010 | 0.99 |
|  | Intervention | Stimulants | 28 | 4.60 | 1.21 | 6.95 | 1.46 | -8.79 | 0.001 |
|  |  | Other types of medication | 25 | 5.87 | 1.42 | 6.79 | 1.26 | -4.09 | 0.001 |

**Table S2** Results of ANCOVA

| Source | Sum of square | df | Mean square | F | P-value | Effect size |
| --- | --- | --- | --- | --- | --- | --- |
| Medication adherence pre-test | 70.03 | 1 | 70.03 | 59.59 | 0.001 | 0.418 |
| Group | 52.35 | 1 | 52.35 | 44.54 | 0.001 | 0.349 |
| Medication type | 6.13 | 1 | 6.13 | 5.21 | 0.025 | 0.059 |
| Group*medication type | 4.77 | 1 | 4.77 | 4.06 | 0.047 | 0.047 |

**Table S3** Parental medication adherence and health literacy by gender

| Variable | Group | Parent | Mean | SD | F | P-value |
| --- | --- | --- | --- | --- | --- | --- |
| Medication adherence | Control | Mothers | 0.06- | 0.89 | -0.869 | 0.391 |
|  |  | Fathers | 0.50 | 0.70 |  |  |
|  | Intervention | Mothers | 1.67 | 1.40 | -0.074 | 0.942 |
|  |  | Fathers | 1.75 | 3.53 |  |  |
| Health literacy | Control | Mothers | 0.12- | 4.09 | -1.13 | 0.264 |
|  |  | Fathers | 3.21 | 0.0 |  |  |
|  | Intervention | Mothers | 13.36 | 6.24 | 0.543 | 0.589 |
|  |  | Fathers | 10.92 | 5.68 |  |  |

**Table S4** Medication adherence and health literacy by education level

| Variable | Group | Educational Status | Mean | SD | F | P-value |
| --- | --- | --- | --- | --- | --- | --- |
| Medication adherence | Control | Non-academic | -0.25 | 1.46 | 0.334 | 0.0801 |
|  |  | Bachelor’s degree | 0.10 | 0.53 |  |  |
|  |  | Master’s degree | 0.00 | 0.15 |  |  |
|  |  | PhD and above | 0.083 | 0.14 |  |  |
|  | Intervention | Non-academic | 1.68 | 1.65 | 0.036 | 0.991 |
|  |  | Bachelor’s degree | 1.72 | 1.48 |  |  |
|  |  | Master’s degree | 1.54 | 1.51 |  |  |
|  |  | PhD and above | 1.55 | 0.99 |  |  |
| Health literacy | Control | Non-academic | 2.28 | 4.42 | 1.89 | 0.152 |
|  |  | Bachelor’s degree | -0.08 | 3.54 |  |  |
|  |  | Master’s degree | -1.74 | 3.42 |  |  |
|  |  | PhD or higher | 0.13 | 4.60 |  |  |
|  | Intervention | Non-academic | 14.94 | 6.33 | 0.518 | 0.672 |
|  |  | Bachelor’s degree | 12.46 | 6.34 |  |  |
|  |  | Master’s degree | 12.79 | 6.30 |  |  |
|  |  | PhD or higher | 13.17 | 5.52 |  |  |

**Table S5** Medication adherence by insurance status

| Variable | Group | Insurance Status | Mean | SD | F | P-Value |
| --- | --- | --- | --- | --- | --- | --- |
| Medication adherence | Control | Under insurance | 0.25 | 0.25 | 0.556 | 0.557 |
|  |  | None | -0.054 | 0.91 |  |  |
|  | Intervention | Under insurance | 2.45 | 1.84 | 1.25 | 0.216 |
|  |  | None | 1.59 | 1.41 |  |  |
